# Implementation of the clinical practice guideline for individuals with amputations in Colombia: a qualitative study on perceived barriers and facilitators

**DOI:** 10.1186/s12913-020-05406-z

**Published:** 2020-06-15

**Authors:** Daniel F. Patiño-Lugo, María del Pilar Pastor Durango, Luz Helena Lugo-Agudelo, Ana María Posada Borrero, Verónica Ciro Correa, Jesús Alberto Plata Contreras, Claudia Yaneth Vera Giraldo, Daniel Camilo Aguirre-Acevedo

**Affiliations:** Facultad de Medicina, Universidad de Antioquia, Grupo de investigación en reahabilitación en salud, Carrera 51 D # 62-29 oficina MUA 302, Medellín, Colombia

**Keywords:** Implementation, Barriers, Facilitators, Clinical practice guidelines, Qualitative research, Colombia

## Abstract

**Background:**

The issue of lower extremity amputation has been in the Colombian political agenda for its relationship with the armed conflict and antipersonnel mines. In 2015 the Colombian Ministry of Health published a national clinical practice guideline (CPG) for amputee patients. However, there is a need to design implementation strategies that target end-users and the context in which the CPG will be used. This study aims to identify users’ perceptions about the barriers and facilitators for implementing the guideline for the care of amputee patients in a middle-income country such as Colombia.

**Methods:**

Semi-structured interviews were conducted with 38 users, including patients, health workers, and administrative staff of institutions of the health system in Colombia. Individuals were purposively selected to ensure different perspectives, allowing a balance of individual positions.

**Results:**

According to participants’ perceptions, barriers to implementation are classified as individual barriers (characteristics of the amputee patient and professionals), health system barriers (resource availability, timely care, information systems, service costs, and regulatory changes), and barriers related to clinical practice guidelines (utility, methodological rigour, implementation flexibility, and characteristics of the group developing the guidelines).

**Conclusions:**

Our study advances knowledge on the perceived individual and health system barriers and facilitators for the implementation of the CPG for amputee patients in Colombia. Importantly, the governance, financial, and service delivery arrangements of the Colombian health system are determining factors in implementing CPGs. For example, the financial arrangements between the insurance companies and the health care provider institutions were identified as barriers for the implementation of recommendations related to the continuity and opportunity of care of patients with amputations. The design of implementation strategies that successfully address the individual behaviours and the contextual health systems arrangements may significantly impact the health care process for amputee patients in Colombia.

## Background

Lower extremity amputation (LEA) is a consequence of disease complications, trauma or accidents [1]. Around 159,000 LEA are performed in the United States each year, most of them are due to diabetes complication [2, 3]. This number will likely increase as a result of the higher incidence of diabetes especially in low- and middle-income countries [4]. In Colombia, according to data from 2010, the prevalence of age-adjusted Diabetes Mellitus type 2 in people over 30 years of age was 7.3% [95% CI (3.7–10.9)] in men and 8.7% [95% CI (5.2–12.3)] in women [5]. LEA data in Colombia is scarce; however, one study conducted in Medellin from 2007 to 2016 with 3.015 patients found that 54% of amputations were due to diseases complication and 46% to trauma [6]. These data coincide with a study in Argentina with 226 amputee patients, 55.6% of whom were due to vascular disease [7]. Also, the Colombian armed conflict has worsened the problem of amputations. According to the Observatory of Antipersonnel Mines in Colombia, 11,801 victims have been registered for antipersonnel mines, between 1990 and 2020, and it is estimated that 70% of those injured mines suffer from limb amputation [8].

Since the year 2010, the Ministry of Health in Colombia funded over 50 evidence-based clinical practice guidelines (CPGs) of priority health problems in the country [9]. Within this initiative, in 2015 our group developed the CPG for the diagnosis, treatment, prosthetic prescription, and rehabilitation of individuals with amputations [10]. However, the efforts to produce high-quality CPGs are not enough to ensure an impact on process and outcomes; consequently, these efforts need to be followed with the design of implementation strategies that target end-users and context in which the CPGs are to be used [11]. The literature acknowledges that the identification of barriers and facilitators is a necessary step to design effective implementation interventions [12, 13]. This is particularly important in low- and middle-income countries where the information about the factors that affect the implementation of evidence-base CPGs is limited [14].

Some authors aimed to clarify how individual (i.e., health provider or patient) and contextual (i.e., health system) factors could operate as barriers to and facilitators of CPGs [15]. At the individual level, the theoretical domains framework (TDF) allows identifying how cognitive, affective, social, and environmental factors influence individual behaviour such as that of health professionals or patients [16]. At the health systems level, it is important to identify how system governance, financial, and service delivery arrangements promote or restrict the options or recommendations endorsed in the CPGs [17, 18].

This research identified the perceptions of potential guideline users, such as patients, health workers, administrators of institutions providing medium- and high-complexity care services, and health insurers, regarding individual and health system barriers and facilitators to implement the CPG for the diagnosis and preoperative, intraoperative, and postoperative treatment, prosthetic prescription, and comprehensive rehabilitation of individuals with amputations.

## Methods

### Setting

Colombia is a high-middle-income country [19] with a social security health system based on regulated competition between private insurance companies providing three affiliation regimes: contributory regime for employees and employers, a subsidised regime for those in need, and special regime for police officers, teachers and others [20]. Also, several health-related insurance options exist, such as supplementary private health insurance, health care insurance for victims of traffic accidents through compulsory traffic accident insurance (SOAT, for its acronym in Spanish), and insurance for work accidents and occupational diseases (ARL, for its acronym in Spanish). In 2008, the Ministry of Health started a process to develop national clinical practice guidelines with economic evaluations and budget impact analysis to inform coverage decision of priority healthcare issues [21].

### Design

A qualitative design was used to explore the perceptions about individual barriers and facilitators and those related to the health system when implementing the CPG for the care of individuals with amputations. Approval was obtained from the ethics committee of the School of Medicine at the University of Antioquia.

### Participants

Thirty-eight individuals were purposefully selected to represent the perspectives of patients, health professionals and managers of health-related institutions. A list of potential key informants was first developed by the study team and subsequently updated by snow-ball sampling technique. Participants included nine amputee patients, six administrative-related professionals from health insurance companies, health care institutions (IPS, for its acronym in Spanish) and non-governmental organisations (NGOs), and 23 health professionals—seven specialists in physical medicine and rehabilitation, four orthopaedists, one general surgeon, two physical therapists, two occupational therapists, two psychologists, one social worker, and four prosthetic technicians. Administrative and health professionals were selected from institutions in four cities in Colombia—Medellín, Cali, Manizales and Bucaramanga—, and patients were selected from Medellin.

### Data collection

Participants were contacted first by telephone and then by a letter, which included an informed consent form, a synopsis of the project, and the guidelines under study. The data collection method consisted of face-to-face semi-structured interviews conducted over 4 months, following a thematic guideline developed from the TDF [16], supplemented with questions on the governance, financing, and service delivery of the health system [17] (see Additional file 1). We conducted pilot interviews within the research group to gain proficiency in conducting the interview and adjust the interview guide.

Interviews were audio-recorded, notes were taken during and after each interview to complement the information. Recordings were transcribed verbatim and the transcriptions were anonymized. Interviews were 42 min long on average. All the data collected were then transferred to the qualitative analysis software NVivo 10 to organise the database and for data coding and analysis.

### Data analysis

Data collection and analysis were interrelated and occurred simultaneously, after training the research team with six workshops, of 4 h each, in the collection, processing, and analysis of qualitative data. The transcribed interviews were analysed by four researchers: DPL, with experience in health systems research, analysed the interviews of administrators; PP, with experience in qualitative research, analysed the interviews of patients and psychosocial professionals; and VC and AP, with clinical experience in rehabilitation and clinical epidemiology, analysed the interviews of clinical health workers. In the first analytical phase, each researcher inductively generated codes reflecting the participants’ perspective and not necessarily the theoretical topics included in the interview guideline. In addition, summaries of participants’ perceptions about barriers and facilitators to guideline use were developed. Later, in team meetings, the codes were grouped into categories and subcategories, which were then described and later related according to their similarities and differences. This allowed the identification of emerging categories based on empirical findings.

Participants were informed as part of the consent process that their names and interview data would be treated confidentially, and the findings anonymized so that they could not be identified. In addition, we asked participants if they were willing to review the transcript of the interview and our interpretation of the data.

It is important to highlight that even though we use a framework form the TDF and the health systems arrangements to develop our interview guide; in the analysis phase we used an inductive approach to generate categories according to the data without following the framework. Therefore, the results are presented according to how we inductively analyzed the emerging categories. In the discussion section, we confront and compare our findings with the theory from the TDF themes and concepts.

## Results

We organized the results according to the barriers and facilitators related to patient characteristics, health professional characteristics, the health care process and those related to the clinical practice guidelines. In each category, the barriers were identified by health care professionals, managers or patients. Additional quotes can be found in the Additional file 2.

### Patient characteristics

The age of the patient, being older, was identified as a barrier for implementing recommendations that were related to their participation in the decision of amputation, the rehabilitation process and the adaptation to the prosthesis. Participants mentioned that the specific characteristics of each patient and the context in which they live should be considered in the recommendation related to an immediate prosthetic prescription. As one participant stated:

*In young patients with traumatic amputation, an immediate prosthesis would be a good option... In older adults, yes, it would be a little more complicated because of all the [conditions], the reason for amputation would make it much more difficult; the rehabilitation process would change dramatically if an immediate prosthetic fitting was made, I think. Health professional*

With patients requiring amputation due to a medical cause, the lack of knowledge of their disease, along with the little or no participation of the patient and their family in decision-making about their amputation, limit the implementation of the guideline’s recommendations.

*Well, many people need it, but I did not ask for it [the amputation] because I knew more or less, it was understood it was what was needed... but many people do not, because losing a lower or upper extremity is hard, but I would recommend not to [be done gradually]; as a diabetic, it has to be amputated immediately. Patient*

Finally, the recommendations related to the rehabilitation, recovery, and adaptation of the patient to the prosthesis are difficult in patients with little autonomy, low self-esteem, and communication deficits.

### Health professional characteristics

Barriers perceived by the participants regarding the provider of amputee care are grouped into those related to their academic training, continuing education, and participation in academic groups organised within the IPS.

The lack of academic training of health professionals in CPGs and evidence-based medicine was identified as a barrier to implementing the CPG. Further, general health practitioners are afraid to deliver some interventions recommended in the CPG for the legal consequences; therefore, they order services and interventions that are not available or unnecessary.

*They [the general health practitioners] are afraid of the legal entity and do not want to do anything that might put them at risk, so, all they do is refer [patients] to the specialist. Manager*

According to the participants, some strategies have been developed that have improved the implementation of the CPG. Some insurance companies develop continuous training programmes for their professionals using distance-education tools. However, these are not specific for the CPG of individuals with amputations but rather for the most prevalent diseases in each institution.

*We have a guideline [example] for diabetes. We test it before, with teams, we modify it, others diagram and edit the guideline and there it is... then we disseminate it and upload it to the web ... We upload it to an educational platform called a virtual classroom... and we monitor the guideline through the audit and quality area [of the institution]. Manager*

The lack of organised academic groups within health care institutions limits the implementation of recommendations. For individuals with amputations, interdisciplinary groups of health and social professionals and prosthetic technicians are important:

*Orthopaedists declared [...] their ignorance about prosthetic management. They said: Yes, we amputate, but of the whole prosthetic part [...] well, about the prosthetic fitting, we have no clue. Health professional*

### Health care process

We identify barriers related to the functioning of the social security health system. The lack of integrated networks between health care institutions of different levels of complexity causes care fragmentation, more administrative procedures, health complications in patients and missed appointments. This also included the lack of an integrated information system for scheduling appointments or setting appointment reminders.

According to participants’ perception, this health care process, fragmented in many cases, is also affected by the type of financial arrangements between insurance companies and the IPS; therefore, patients must attend different institutions to receive the services they need. Prompt payments to the IPS also affect timely care. Some participants, especially physiatrists, acknowledge that patients receive better care, more timely care and faster prostheses delivery, when they have private health insurance plans or are affiliated with ARL, or when the care costs are assumed by SOAT or an NGO. The following testimony illustrates differential care depending on the type of health insurance.

*... some require the diagnostic help of the group, but almost all trauma cases are traffic accidents, so their process is much faster... since SOAT is, in theory, a good payer, those go fast; if one says that is a SOAT patient for amputation or anything else, they get care quickly, and diagnostic aid is also quickly available. Manager*

As a facilitator, we found that insurance companies audit health care provider institution in their compliance with CPGs. Also, in some institutions, adherence to the guidelines is part of the performance evaluation which includes the review of some medical records checking for compliance with CPG recommendations.

*Two Wednesdays a month we have a medical records committee, and in one, we measure and evaluate adherence [to the CPG]; then, I look for patients with traumatic brain injury as this is the condition we are measuring at the time, and then, with the checklist, we verify whether what they did, let's say the physiotherapist, is fulfilling the objectives according to the checklist. Manager*

Frequent regulatory changes in the health system hinder service delivery, posing barriers to implementing the CPG by creating uncertainty about patients’ rights according to the contents of the benefit plan.

*For example, national policies and the guidelines should be clear because I must know what this patient is entitled to, and although health institutions are working on the rights and duties of patients with disabilities, we continue to ignore many of those rights... Health professional.*

According to the participants, insurance companies sometimes wrongly assess the cost-effectiveness of prosthetic components and assume that lower costs of prosthetic prescriptions generate more savings. These leads patients to resort to legal claims so their right to care is respected.

The location of service delivery was also important for the implementation of the recommendations. Patients living in rural areas with scarce economic resources have problems attending service sites due to transportation costs and logistical difficulties when requiring someone to accompany them. In rural areas, there are neither interdisciplinary teams nor prosthesis manufacturing workshops as require by some recommendations of the CPG.

### Clinical practice guidelines

Study participants acknowledge barriers related to the technical concepts within the guideline, the design of the CPG document, the characteristics of the group developing the guidelines, and the dissemination process. Regarding the technical concepts within the guideline, for many, the relationship between levels of evidence and the strength of the recommendation is not clear, specifically when low levels of evidence may lead to a strong recommendation or high levels to a weak recommendation. This confusion generates uncertainty as to the binding force of the recommendations, whether they are mandatory or flexible in their application.

*I am surprised by the low levels of evidence. Everyone consults guidelines with good levels of evidence; low evidence-based guidelines might be thought as not strong enough to support clinical practice, providing greater variability to the medical act than those with higher levels of evidence. Manager.*

Participants value the involvement of academics in the groups developing guidelines, ensuring an adequate method and a rigorous research process, which implies the guidelines are valuable educational material. Three important characteristics are acknowledged: experience in developing CPGs, participants’ specific thematic expertise, and skill in the management and care of patients.

Regarding the content and presentation of the guidelines, participants perceive them as impractical, dense and extensive; therefore, the doctors do not apply them. Some participants perceive a lack of dissemination of the guidelines for the care of individuals with amputations, constituting a barrier to implementation, since few users are familiar with it, possibly because of its novelty, the scarcity of dissemination by the Ministry of Health, or the quality of the institutional induction and reinduction processes for the professionals responsible for patient care, as expressed by a participant:

*...The other point is to disseminate the guideline, it should be accessible to people so they can read it; I reviewed practically everything; it seems to me it is clearly written, in a very accessible language. Specifically, in what concerns us in prosthetic rehabilitation, I like it clarifies prescription possibilities, depending on the level of amputation and, at the same time, the level of the functionality of each patient. Health professional*

The lack of credibility of local guidelines constitutes an additional barrier to implementation since, for some participants, the specialists rely more on the evidence reported on CPGs published in other countries, or sometimes guidelines of the pharmaceutical industry or medical device manufacturers than on the guidelines developed in their own country.

The guidelines can include strategies to use resources more efficiently and achieve better outcomes for patient care, such as fewer infections, less hospitalisation, or faster recovery. Some participants mentioned possible barriers to implementation if the benefits are not clear and the guidelines recommend expensive technology. For example, the recommendation of an immediate prosthesis is perceived as one of the most difficult to implement because it requires the coordination of multiple stakeholders. The possibility of a double cost for the insurance company is also mentioned: payment of both the temporary and permanent prostheses.

Another barrier to implementation is related to the inclusion of products and services recommended in the guidelines in the health benefit plans and their availability in different regions of the country. Participants perceive a lack of coordination between the scientific and technical components of the guidelines and their implementation by the Ministry of Health.

## Discussion

To our knowledge, this is the first study to assess barriers and facilitators of CPG related to amputee patients in a low- and middle-income country. This research allowed the identification of individual and health system barriers for implementing guidelines for individuals with amputations; the barriers were related to the characteristics of patients, professionals, the health care process, and CPG in Colombia. For the following discussion, we grouped the barriers according to the TDF and health system arrangements framework, then we mapped those barriers to a behavioural system, the COM-B system, in order to identify which TDF domains are important in changing behaviour and that could be the target of future interventions [22, 23].

### Theoretical domains: individual barriers

#### Knowledge and skills barriers

The lack of knowledge and skills by health workers was one of the main barriers encountered. Of these conditions, not knowing of the existence of guidelines or not being familiar with their content has been reported as a barrier in different studies [24–27] including studies conducted in low and middle-income settings [28]. According to the COM-B system, knowledge and skills are part of the individual phycological capacities needed to engage in specific activities [23]. Therefore, educational and training interventions that aim at increasing knowledge or understanding and imparting skills could be considered to address these barriers [22]. Intervention designers should take into account the evidence from low and middle-income countries that shows how quality improvement activities, including education interventions, may be affected by workplace culture characteristics like trust and intrinsic health care professional motivation [29].

#### Barriers related to beliefs about capabilities and consequences

The results of the present study also show a generalised perception of the lack of ability of general practitioners to follow guideline recommendations because of three main factors: little information about patients with amputations and prosthetics, lack of material and human resources in municipalities, and fear of legal consequences for treating patients without the direction of a specialist; these issues have also been reported as a barrier to implementation in other studies [27, 30]. This domain also refers to the capacity of patients to take part in decision-making, the communication between the physician and the patient, and the autonomy and self-esteem of the patient.

Barriers related to beliefs about capabilities and consequences can be classified under the motivation component of the COM-B system [23]. The motivation component includes the brain process that direct behaviour like emotions, social identity, goals and plans [22]. These barriers can be addressed with interventions aiming to increase knowledge and elicit positive feelings about the desired behaviour through persuasion or incentivisation [22]. In low and middle-income countries, high intrinsic health care professional motivation seems to be an enabler of successful guideline implementation [29] and could be influenced by financial incentives to apply the recommended care [31].

### Health system arrangements

The governance, financial, and service delivery arrangements of the Colombian health system are determining factors in implementing CPGs. These factors are considered under the social or physical opportunity component of the COM-B system, which can be defined as the outside factors that make a specific behaviour possible, like the external environment, resources, time, social factors, cultural norms [22]. Intervention to address these factors includes restriction, environmental restructuring to change the social or physical context and enablement [22].

#### Governance arrangements

We found rules and processes that create a favourable environment for the implementation of CPG in Colombia. The regulations of health services in Colombia require health care institutions to have procedures for developing or adapting CPGs about the most prevalent health conditions encountered in the institution. These guidelines must be evidence-based, and the institutions can adopt the CPGs developed by the Ministry or develop their guidelines following the methodological manual of the Ministry of Health. In addition, the staff of the institution must be familiar with the guidelines [32].

National policies and legislation that intended to ensure quality, reduce variability, and establish work performance standards have been important mechanisms for promoting the implementation of CPGs in Colombia and other context [30, 33]. However, a systematic review with a focus on developing countries identified that leadership, governance and policy-related issues are substantial barriers in the successful implementation of misoprostol use for post-abortion care and post-partum haemorrhage prevention [34].

#### Financial arrangements

Although some studies have mentioned economic and financial factors as barriers to implementing guidelines [33, 35], few have described the financial aspects of health systems based on regulated competition. Here, the financial arrangements of the health system, specifically, the agreements between payers and providers, affect the implementation of recommendations. In Colombia, those responsible for payment of health services are health insurers, Health Secretariats, Adapted Entities, and transit insurance; and the opportunity of care is sometimes affected by the institutions that pay for the service.

#### Service delivery arrangements

Regarding service delivery arrangements, it was found that the service site, the training of service providers, and their performance evaluation affect guideline implementation.

The findings regarding the location of service delivery are consistent with some studies reporting that the characteristics of the work environment—such as working in rural areas, during night shifts, or with little interaction with other doctors—negatively affect the implementation of recommendations [26].

When comparing health systems barriers between high and low-income countries differences are mainly in terms of availability of health care resources like lack of space, equipment, and shortage of staff were [31]. Also, evidence has also shown that barriers in low-income countries are due not only to limited resources but weak health systems, limited organizational capabilities and poor management of existent resources [36].

Some characteristics of CPGs as support documents to provide health services limit their implementation. Some participants consider the terminology for levels of evidence levels and strength of recommendation complex, the guidelines extensive, and some recommendations difficult to implement, with possible additional costs for insurance companies. Other studies have indicated that the perceived complexity of the guidelines or of the specific recommendations limits their implementation [25, 37, 38].

Perceived characteristics of guidelines positive for implementation were also found. As some have noted [38], the credibility of the experts who develop the guidelines and patients’ involvement in this process favour implementation. However, some participants preferred international guidelines to national guidelines, which is difficult to interpret when considering the perceptions about the participants in the group developing the guidelines and the adaptation of the guidelines to the local context.

### Strengths and limitations

The present study reveals some barriers and facilitators for implementing CPGs identified by different stakeholders of the Colombian health system. This information, scarce in Colombia, is necessary to ensure that effort initiated over 10 years ago has a better chance of positively impacting the health of people. The possibility of interviewing people from different areas of health care and different institutions and cities allowed the creation of a coherent narrative, identifying specific barriers to an insurance-based health system and providing important contributions to the international literature.

In the present study, data collection and the discussion of the findings were guided by the TDF [16] and health system arrangements [17]. This is important because some authors have expressed the need to make explicit use of the theory to identify barriers and facilitators and to design and implement interventions to overcome these barriers [39–42].

The present study has some limitations. In some instances, the study yielded general responses because of the lack of knowledge of this CPG by some participants. Participants were recognised professionals in the care of individuals with amputations and work in cities with resources to care for these patients, which can be a limitation to identify barriers for implementing guidelines in rural areas with greater resources limitation.

### Implications for practice, research, and policy

The barriers and facilitators found in the present study should be considered to design implementation strategies sensitive to the Colombian institutional context. For example, to overcome the barrier of lack of knowledge and skills for implementing guidelines for individuals with amputations, guideline developers and researchers interested in their implementation can develop educational courses using distance-education strategies for professionals located in different municipalities of the country. Regarding system governance, the enabling regulations for health services in Colombia may require institutions to establish procedures to adapt CPGs to all conditions encountered, not only the most prevalent. From an equity perspective, it makes little sense for the health system to prioritise the integration of care based on scientific evidence to reduce unjustified clinical variability and to improve clinical outcomes only for some conditions and not for all.

Regarding the challenges imposed by the barriers related to health care financing and insurance, the task for policymakers is to ensure that access to the promotion, prevention, diagnosis, treatment, and rehabilitation of individuals with amputations does not depend on the type of patient insurance. This issue has been addressed in academic articles [43, 44] and news media [45]; however, the solutions are still not very clear.

Finally, the developers of CPGs can help overcome the barriers of complex guidelines and difficulty in understanding technical terminology, such as that for levels of evidence and strength of recommendation, developing checklists with interventions and key practices at specific care times and places. The methodological rigour with which the CPGs are developed in Colombia must continue, but the final products must improve. For example, a checklist to confirm who should do what, where, and at what time can be useful for improving adherence to recommendations [46].

## Conclusions

Our study advances knowledge on the perceived individual and health system barriers and facilitators for the implementation of the CPG for amputee patients in Colombia. Importantly, the governance, financial, and service delivery arrangements of the Colombian health system are determining factors in implementing CPGs. For example, the financial arrangements between the insurance companies and the health care provider institutions were identified as barriers for the implementation of recommendations related to the continuity and opportunity of care of patients with amputations. The design of implementation strategies that successfully address the individual behaviours and the contextual health systems arrangements may significantly impact the health care process for amputee patients in Colombia.

## Supplementary information

**Additional file 1.** Thematic guidelines for interviews.

**Additional file 2.** Additional quotes.

## Data Availability

To request the data used and analysed for the present study, contact the first author, justifying your request, as it involves transcripts of interviews, and anonymity and confidentiality must be maintained.

## Abbreviations

CPG: Clinical practice guideline
LEA: Lower extremity amputation
TDF: Theoretical domains framework
SOAT: Health care insurance for victims of traffic accidents
ARL: Insurance for work accidents and occupational diseases
IPS: Health care institutions
NGOs: Non-governmental organizations
