# Supplementary material for: Implementation of the clinical practice guideline for individuals with amputations in Colombia: a qualitative study on perceived barriers and facilitators
Source: BMC Health Serv Res. 2020 Jun 15;20:538. doi: 10.1186/s12913-020-05406-z (PMC7296745; doi:10.1186/s12913-020-05406-z)
Supplement: Supplementary file 1 — Additional file 1. Thematic guidelines for interviews. [file 12913_2020_5406_MOESM1_ESM.docx]

**Additional file: Thematic guidelines for interviews.**

1. **For patients**

This interview aims to “Identify the perceptions of CPG users (patients, caregivers, health workers, institutions providing II and III level of care and health promoting companies), about facilitators and barriers to their implementation." Therefore, the important thing is to listen to the participants and keep in mind that perceptions are the meanings and the sense that people (social subjects) give to reality based on their own history, interactions and the sociocultural environment.

It is intended rather than asking questions and waiting for answers, it is important to have a fluid conversation with the participant in which the different issues of interest for this research are addressed, therefore it is not about applying a questionnaire. It is suggested to follow an order in the conversation and avoid asking questions about matters that were answered at an earlier time. This guide is flexible and changes depend on the analysis of each interview, so that new questions or topics can be included as the information gathering progresses.

The questions are adapted from Michie 2005, the Ministry handbook and from Lavis 2002.

Hints for the interviewer are in parenthesis or with the symbol •

**Questions**

1. Please tell us, how was your experience when you were amputated (reconstruction and amputation in two stages, access to services, those who attended you, care by an interdisciplinary team, time elapsed between the indication of amputation and its performance).
2. Tell us about your experience before your amputation, the conversation you had with the doctor to define the level of amputation, the care by a psychologist, participation in the decision, how they informed you about the procedure they were going to do.
3. Tell us about the process you had to carry out to obtain the prosthesis, (access to services, those who attended you, care by interdisciplinary team, time elapsed between amputation and getting the prosthesis and between prostheses and its adaptation, how was your participation in the decision regarding the type of prosthesis).
4. How has been your rehabilitation process and integration to your daily activities? How have your caregivers been linked to this rehabilitation process?
5. What have been the positive and negative consequences for you due to the process of amputation and adaptation to the prosthesis? (costs for displacement, disability, care, rehabilitation, prosthetics).
6. What changes would you make to the care process: before, during and after the amputation?
7. Tell us about your experience in the health care insurance (EPS) before during and after the amputation. (consequences of caring for amputees in the EPS).
8. In which cases, you or other patients have used legal claims to achieve care during the amputation, prosthetics and rehabilitation process?
9. What have you heard about Clinical Practice Guidelines for amputee care?

• Show the CPG recommendations and based on these, ask:

1. What recommendations do you think are convenient for you, the person you care for, or other patients?
2. What would have to be changed or made different to carry out the recommendations of this CPG (new behaviors or changes in existing behaviors)?

12. What do you think that will be the consequences (positive and negative) for you, other patients, health professionals, organizations – health insurance EPS- health care institutions IPS, if these recommendations are implemented, including costs?

13. What do you think that will happen if these recommendations are not implemented?

14. Do you know other people who have carried out activities related to these recommendations?

15. Do you know institutions where these recommendations are being followed?

16. What situations facilitate or limit compliance with these recommendations at the site where you are attended?

• Consider the following topics during the conversation with the patient, although they could have already been answered.

17 What recommendations can cause financial difficulties (payment of moderating fees, copays or out-of-pocket expenses, transfers)?

18. What do you consider to be the costs for IPS / EPS and what are the costs of the implementation these recommendations?

19. How do you see the relationship between EPS and IPS for the implementation these recommendations?

20. Is it easier to comply with these recommendations with prepaid medicine plans (private health insurance)?

21. What are the differences between subsidized, contributory, special regimes and none insurance options for the attention of amputee person?

Service design

• Explore whether the guideline recommendations are available in the IPS / EPS for all patients who need them in a timely manner and whether there are enough professionals and technicians to carry it out.

Service location

• Explore whether the location where the service is provided affects the implementation of the recommendations. Think about areas of difficult access and the differences in the application of the recommendations according to the site of first care for the patient.

Existence of infrastructure and equipment necessary to implement the recommendations.

Aid for the provision of the service.

• Operation of the information system for appointment reminders and electronic medical records.

1. **For health professionals**

This interview aims to “Identify the perceptions of CPG users (patients, caregivers, health workers, institutions providing II and III level of care and health promoting companies) about facilitators and barriers to their implementation." Therefore, the important thing is to listen to the participants and keep in mind that perceptions are the meanings and sense for that people (social subjects) give to reality based on their own history, interactions and the sociocultural environment.

It is intended that rather than asking questions and waiting for answers, a fluid conversation with the participant is had in which the different issues of interest for this research are addressed, therefore it is not a question of applying a questionnaire. It is suggested to follow an order in the conversation and avoid asking questions about matters that were answered at an earlier time. This guide is flexible and changes depend on the analysis of each interview, so that new questions or topics can be included as the information gathering progresses.

The questions are adapted from Michie 2005, the Ministry handbook and from Lavis 2002

Some questions have endnotes in parentheses that highlight important issues to consider.

Some questions instead of having numbers have bullets to indicate that the answers to these questions may have arisen in previous questions.

In this guide, the system “The Grading of Recommendations Assessment, Development and Evaluation (GRADE) ”was used to rate the evidence and grade the recommendations.

| Quality of Evidence | | | |
| --- | --- | --- | --- |
| High | We are very confident that the true effect lies close to that of the estimate of the effect.  Further research is very unlikely to change our confidence in the estimate of effect. | **⊕⊕⊕⊕** | **A** |
| Moderate | We are moderately confident in the effect estimate: The true effect is likely to be close to the estimate of the effect, but there is a possibility that it is substantially different.  Further research is likely to have an important impact on our confidence in the estimate of effect and may change the estimate. | **⊕⊕⊕○** | **B** |
| Low | Our confidence in the effect estimate is limited: The true effect may be substantially different from the estimate of the effect.  Further research is very likely to have an important impact on our confidence in the estimate of effect and is likely to change the estimate | **⊕⊕○○** | **C** |
| Very low | We have very little confidence in the effect estimate: The true effect is likely to be substantially different from the estimate of effect.  Any estimate of effect is very uncertain | **⊕○○○** | **D** |

The strength of a recommendation is rated in four categories: **Strong or Weak for** use of the intervention or **Strong or Weak against** of the intervention

**Strong:** Most informed people would agree to the recommended action, only a small proportion would not. Recommendations can be accepted as a health policy in most cases.

**Weak:** Most informed people would agree to the recommended action, but a significant number would not. Values and preferences can vary widely. The decision as a health policy merits important debate and discussion with all interest groups.

**Questions**

Barriers and Individual Facilitators

Knowledge

1. Tell me a little about your experience with people who require lower limb amputation (It is important to know in which institutions the professionals work and have worked).
2. What Clinical Practice Guidelines have you used during your professional performance?
3. Which of the Clinical Practice Guidelines that the Ministry of Health has developed do you know?
4. What recommendations do you know from the CPG for preoperative, intraoperative and postoperative diagnosis and treatment of the amputated person, the prescription of the prosthesis and comprehensive rehabilitation, developed by the Ministry of Health? (What recommendations did you not know? ¿How would you define the evidence? The second part of the question was probably included in the answer to the first part.
5. What would you modify of the recommendations that XXXX (briefly state the recommendations that are prioritized for the professional interviewed)? What characteristics should the professionals who carry out these recommendations have? Explain your reasons.

Skills

1. When do you think you can start implementing recommendations XXXX? briefly remember the recommendations sent? Explain a bit the reasons for your answer.
2. In what situations are compliance with these XXX recommendations facilitated or hindered in your workplace? (Identify if they cannot comply with the recommendations because they are not capable, they do not have the training, there are differences what they do in their daily practice).

Social and professional identity

1. What opinion do you have about the people who made the guideline?
2. What do you think you should know about these people? (Identify aspects related to the credibility of those who made the guide, on the credibility of national vs. international guides) This question will probably be answered with the previous question?
3. What do you consider to be the usefulness of this CPG (for diagnosis and preoperative, intraoperative and postoperative treatment of the amputated person, the prescription of the prosthesis and comprehensive rehabilitation)? This question is likely answered in the previous question.
4. What do you consider to be the advantages for professionals, patients and institutions? (The questions about the usefulness and advantages are intended to identify whether professionals consider that the guidelines should determine their behavior, if doing what the recommendations describe is compatible with professional standards, identify moral, ethical issues, limits to autonomy).

Beliefs about capabilities and consequences

1. What would you do to start the implementation of the CPG in the institution where you work? (Identify what problems have arisen to follow the recommendations, which can help to make the recommendations).
2. What do you think that will happen after implementing the recommendations of the XXXX guide? (How long do these consequences occur in the short or long term, what are the costs and benefits of these consequences).
3. What do you think that will happen if the recommendations are not implemented (e.g., recommendation 3, 4, 12, 21, 43)?

Recommendation 3: Coverage with plastic surgery vs. raise the level of amputation

Recommendation 4. Reconstruction vs. Amputate

Recommendation 12. Preoperative psychological support

Recommendation 21. Myodesis vs. myoplasty in transfemoral amputation

Recommendation 43. Comprehensive rehabilitation in amputees vs. usual care

• How would you feel if these recommendations are implemented or not?

Motivation and goals

1. How much do you agree with the implementation of these recommendations?

• How much do you think it is necessary to implement these recommendations?

1. What issues motivate you to comply with the recommendations? (incentive system).

Memory, attention and decision process

1. Which of the recommendations do you usually meet?
2. Which of the recommendations you may decide not to follow? Explain the reasons (time, costs, customs, other evidence, another school ...)

Social influences

1. Who do you think influences the implementation of the recommendations and who could limit their implementation? (medical colleagues, specialists, other health professionals, managers, executives, patients, caregivers, opinion leaders, professional associations, training institutions for health professionals, IPS)
2. Which colleagues or institutions that you know, comply with these recommendations?

Nature of behavior

1. What would have to be changed or made different to carry out the recommendations of this CPG (new behaviors or changes in existing behaviors)?

Barriers or facilitator of health system

1. When, where, how, how often and with whom could recommendations or changes be made? (Barriers and facilitators at the health system level). Note: Not all participants will be able to answer these questions, but some will likely mention how health system arrangements influence the implementation of the recommendations.

Political authority

1. What existing regulations in the country do you know that contribute to promoting the implementation of the recommendations?

Organizational Authority

1. How can the legal nature of IPS or EPS affect the implementation of the CPG? It refers to whether they are public or private. Explore whether profit making can affect implementation
2. What is the interest that organizations may have in leading CPG implementation processes? (incentives regarding accreditation or authorization of services, reduction of costs of care).

Financing the system

1. What recommendations can generate financial difficulties for the IPS or for the patient (payment of moderating fees, copays or out-of-pocket expenses, transfers of patients)?
2. How do you see the relationship between EPS and IPS for the implementation of these recommendations?

(Is it easier to comply these recommendations in some EPS or IPS with prepaid medicine. Differences between subsidized, contributory, special and none insurance regimes)

Funding of organizations.

Type of hiring and remuneration of professionals and technicians: Salary, fee for services, per capita. Purchase of products and services. Glosses).

1. Does the inclusion in the POS facilitate the implementation of these recommendations? (Explore limitations or difficulties, Glosses)
2. In which cases have patients resorted to legal claims to achieve care?

The following topics may have been answered in previous questions. Please review and supplement if necessary:

Service design

• Explore whether the guideline recommendations are available in the IPS / EPS for all patients who need them in a timely manner and whether there are enough professionals and technicians to carry it out.

• Explore whether the location where the service is provided affects the implementation of the recommendations. Think about areas of difficult access and the differences in the application of the recommendations according to the site of first care of the patient.

• Existence of infrastructure and equipment necessary to implement the recommendations.

Aid for the provision of the service.

• Information system for monitoring and evaluating the implementation of the CPGs; reminders and prompts, electronic medical records, quality monitoring systems.

1. **For managers:**

This interview aims to “Identify the perceptions of CPG users (patients, caregivers, health workers, institutions providing II and III level of care and health promoting companies) about facilitators and barriers to their implementation." Therefore, the important thing is to listen to the participants and keep in mind that perceptions are the meanings and sense for that people (social subjects) give to reality based on their own history, interactions and the sociocultural environment.

It is intended that rather than asking questions and waiting for answers, a fluid conversation with the participant is had in which the different issues of interest for this research are addressed, therefore it is not a question of applying a questionnaire. It is suggested to follow an order in the conversation and avoid asking questions about matters that were answered at an earlier time. This guide is flexible, and changes depend on the analysis of each interview, so that new questions or topics can be included as the information gathering progresses.

The questions are adapted from Michie 2005, the Ministry handbook and from Lavis 2002

Some questions have endnotes in parentheses that highlight important issues to consider.

Some questions instead of having numbers have bullets to indicate that the answers to these questions may have arisen in previous questions.

In this guide, the system “The Grading of Recommendations Assessment, Development and Evaluation (GRADE) ” was used to rate the evidence and grade the recommendations.

| Quality of Evidence | | | |
| --- | --- | --- | --- |
| High | We are very confident that the true effect lies close to that of the estimate of the effect.  Further research is very unlikely to change our confidence in the estimate of effect. | **⊕⊕⊕⊕** | **A** |
| Moderate | We are moderately confident in the effect estimate: The true effect is likely to be close to the estimate of the effect, but there is a possibility that it is substantially different.  Further research is likely to have an important impact on our confidence in the estimate of effect and may change the estimate. | **⊕⊕⊕○** | **B** |
| Low | Our confidence in the effect estimate is limited: The true effect may be substantially different from the estimate of the effect.  Further research is very likely to have an important impact on our confidence in the estimate of effect and is likely to change the estimate. | **⊕⊕○○** | **C** |
| Very low | We have very little confidence in the effect estimate: The true effect is likely to be substantially different from the estimate of effect.  Any estimate of effect is very uncertain | **⊕○○○** | **D** |

**Questions**

**Barriers and facilitators Individual**

Knowledge

1. What do you know about the Clinical Practice Guidelines that the Ministry of Health has developed?
2. What do you know about the Guideline to Clinical Practice of diagnosis and preoperative, intraoperative and postoperative treatment of the amputated person, the prescription of the prosthesis and comprehensive rehabilitation, developed by the Ministry of Health? What recommendations did you not know?
3. What is an evidence-based clinical practice guideline for you? How would you define the evidence?
4. What do you think about the recommendations we send you? Who should carry out these recommendations? Explain your reasons.

Skills

1. When do you consider that these recommendations can be implemented in your IPS / EPS / ONG? Explain a bit the reasons for your answer.
2. What situations facilitate or limit compliance with these recommendations on the site where you work? (Identify if you respond to moral, ethical, legal issues, emotions, reactions, limits to autonomy, if you cannot comply with the recommendations because you do not have the training, there are differences with what you do in your daily practice)

Social and professional identity

1. What do you know about the people who made the guideline?
2. What do you think you should know about these people?
3. What do you consider to be the usefulness of this CPG (Clinical Practice Guideline for diagnosis and preoperative, intraoperative and postoperative treatment of the amputated person, the prescription of the prosthesis and comprehensive rehabilitation)? What are the advantages for professionals, patients and institutions?

Beliefs about consequences

1. What must be done to start the implementation of the CPG?
2. ¿What do you think will be the consequences (positive and negative) for patients, colleagues, organizations –EPS-IPS-SGSSS if the recommendations we send you are implemented? When these consequence could occurre? (short and long term).
3. What do you consider to be the costs they represent for the IPS / EPS / for the system?
4. What do you think will happen if these recommendations are not implemented? Identify or explore the cost / benefit ratio of implementing the recommendations.
5. How would you feel if these recommendations are implemented or not?

Motivation and goals

1. How much do you agree with the implementation of these recommendations?
2. How much do you think it is necessary to implement these recommendations?
3. What issues motivate you to comply with the recommendations? (incentive system)

Memory, attention and decision process

1. Which of the recommendations are usually met in your institution?
2. Which of the recommendations may you decide not to follow at your institution? Explain the reasons (time, costs, custom, other evidence).

Social influences

1. Who do you consider influencing compliance with the recommendations and who could limit their implementation? (doctors, specialists, other health professionals, managers, auditors, managers, patients, caregivers, opinion leaders, professional associations, training institutions for health professionals).
2. In which institutions that you know are these recommendations met?

Nature of behavior

1. What would have to be changed or done differently in the IPS / EPS / SGSSS to carry out the recommendations of this CPG (new behaviors or changes in existing behaviors)?.

Barriers and facilitators at the health system level

1. When, where, how, how often and with whom could recommendations or changes be made? (Barriers and facilitators at the health system level). Note: Not all participants will be able to answer these questions, but some will likely mention how health system arrangements influence the implementation of the recommendations.

Political authority

1. What policies or regulatory standards promote the implementation of the recommendations?

Organizational Authority

1. How can the legal nature of the IPS or EPS affect the implementation of the CPG? It refers to whether they are public or private. Explore whether profit making can affect implementation.
2. What is the interest that organizations may have in leading CPG implementation processes? (incentives regarding accreditation or authorization of services, reduction of costs of care).

Financing the system

1. What recommendations can generate financial difficulties for the IPS or for the patient (payment of moderating fees, copays or out-of-pocket expenses, transfers of patients)?
2. How do you see the relationship between EPS and IPS for the implementation of these recommendations?

(is it easier to comply with these recommendations in some EPS or IPS with prepaid medicine).

Consider: differences between subsidized, contributory, special and uninsured regimes Funding of organizations. Type of hiring and remuneration of professionals and technicians: salary, fee for services, per capita. Purchase of products and services.

1. Does the inclusion of these recommendation in the health benefit plan facilitate their implementation? Explore limitations or difficulties.
2. In which cases of the recommendations of this CPG, have patients resorted to legal claims to achieve care?

The following topics may have been answered in previous questions. Please review and supplement if necessary:

Service design

• Explore whether the guideline recommendations are available in the IPS / EPS for all patients who need them in a timely manner and whether there are enough professionals and technicians to carry them out.

Service location

• Explore whether the location where the service is provided affects the implementation of the recommendations. Think about areas of difficult access and the differences in the application of the recommendations according to the site of first care of the patient.

Existence of infrastructure and equipment necessary to implement the recommendations.

Aid for the provision of the service.

• Operation of the information system for monitoring and evaluating the implementation of the CPGs; for reminders, electronic medical records, quality monitoring systems.

**Definition the terms:**

1. CPG: Clinical Practice Guideline
2. EPS: Health Insurance
3. IPS: Care providers Institutions
4. POS: Mandatory Health Plan
