# Supplementary material for: Implementation of the clinical practice guideline for individuals with amputations in Colombia: a qualitative study on perceived barriers and facilitators
Source: BMC Health Serv Res. 2020 Jun 15;20:538. doi: 10.1186/s12913-020-05406-z (PMC7296745; doi:10.1186/s12913-020-05406-z)
Supplement: Supplementary file 2 — Additional file 2. Additional quotes. [file 12913_2020_5406_MOESM2_ESM.docx]

Additional file # 2: Additional quotes

| **Implementation**  **barriers** | **Description** | **Quote** |
| --- | --- | --- |
| Patients | Limitations in communication. | ***“***If you do not express yourself, or if it is difficult for you to express yourself, the diagnosis will not be correct, that is why I tell you that there could be very good professional in psychology, in psychiatry, but if you do not have the way to communicate what you are really feeling, they will not guide you. So I think it goes beyond your awareness, where you want to get to, what you want to do, want to get up or not, want to be a burden or not" (Patient) |
|  | Geographical barrier  Place of residence | "Well, for the patients who still consult, victims of antipersonnel mines, a great barrier is the geographical barrier, they are in very remote areas, and the costs of transportation, the logistics of accommodation and stay in the city to do the paperwork, they don't always have it at hand. " (Professional) |
| Professionals | Articulation of academic and practical training in University hospitals | "... In this hospital, in all departments there are people from the university working on guidelines, they are hospital guidelens, but the university is participating in the processes of construction, adaptation, and adoption of those for the hospital, that is, the role that the university plays is of first order. " (Professional) |
|  | Multidisciplinary work. Lack of continuity in the comprehensive care process | "The patients who had amputation, mainly traumatic, spend months without having a prosthetic adaptation, so there was an initiative from the orthopedic department to do a staff to see if the group meeting could put more pressure, so that these patients adapted more quickly, and in addition, the orthopedists expressed […] their ignorance about all prosthetic treatment . They said: “Yes, we do all the amputation, but the entire prosthetic process […] well, prosthetic adaptation we have no idea. " (Professional) |
| Health care sevices | Need for interdisciplinary work.  Difficulty in communication between different professionals | No institution, whatever it may be, can modify any medical prescription, none, unless there is some other commitment in that user, there are times when a prosthesis or an orthopedic aid may have been badly prescribed, sometimes it happens, and for example, as a technician... here in Cali we have that possibility, we communicate with the doctors: " doctor, I think this knee is not the right one for this patient" so what happens at that moment?. We make a new appointment to the user, it is reviewed again and if there is a need to do a new prescription, it is done ... "(Professional) |
|  | Continuity of care.  Information system that articulates the different levels of care with users (patients) | We have a new patient who is entering the in the care pathway, he started two months ago, at this moment he is in the authorization process, he has already made the route, he went to the general doctor in that EPS, then general doctor sent him to the orthopedic doctor , and this doctor sent him to the physiatrist, finally this doctor made his prescription and it is already in the process in the EPS for authorization; so it´s been two months, let's say it went well. Let's see how long it takes… ”(Professional) |
|  | Difficulty of access for prostheses for patients on the subsidized regimen  Little opportunity in the delivery of prostheses for patients of the subsidized regime | “These patients generally belong to the subsidized regime or are patients with inability to pay. These are the ones that are served by the ICRC. The ICRC brings them, and they are the ones who, in accordance with the agreement we have with the hospital, facilitate the entire process of patient rehabilitation” (Professional) |
| Clinical Practice guideline | Articulation of universities and health sector institutions to facilitate implementation of guidelines | "Within the agreement [between the university hospital and the university], they put on the heads of department chief, the adoption and adaptation of the guidelines [...] in the hospital we have guidelines, we must take the summary guide, and adapt it to a reference document that is already established by the document management department, so we are already working […] and the idea is that as soon as the amputee is published, we will do the same process, but it is already established how it will be done" (Professional) |
